# Cortisol Awakening Response and Stress in Female Nurses on Monthly Shift Rotations: A Longitudinal Study

**DOI:** 10.1155/2022/9506583

**Published:** 2022-09-15

**Authors:** Yu-Huei Lin, Hsiu-Ju Jen, Yen-Kuang Lin, Jang-Dong Seo, Wen-Pei Chang

**Affiliations:** ^1^Post-Baccalaureate Program in Nursing, College of Nursing, Taipei Medical University, Taipei, Taiwan; ^2^Department of Nursing, Shuang Ho Hospital, Taipei Medical University, New Taipei City, Taiwan; ^3^Graduate Institute of Athletics and Coaching Science, National Taiwan Sport University, Taoyuan, Taiwan; ^4^Department of Statistics, Indiana University, Bloomington, USA; ^5^Department of Nursing, Shuangho Hospital, Taipei Medical University, New Taipei City, Taiwan; ^6^School of Nursing, College of Nursing, Taipei Medical University, Taipei, Taiwan

## Abstract

The majority of shift nurses are female, there is still an expectation that they fulfil the traditional role of women in the family in Asia, often conflicting with shift work, increases stress, and affects cortisol secretion patterns. This study was to understand the changes in the cortisol awakening response (CAR) and work stress in nursing personnel working in different shifts. We recruited 41 female shift nurses. We administered the Taiwan Nurse Stress Checklist (NSC), and the nurses themselves collected saliva samples upon waking and 30 minutes after waking for three consecutive days at home. The saliva samples enabled us to analyze the increase in cortisol levels following waking (CARi) of nurses working different shifts (day, evening, and night). We then analyzed the data obtained using a hierarchical linear model (HLM). The results indicated that in terms of stress from the inability to complete personal tasks, the regression coefficients of night-shift nurses vs. day-shift nurses (*B* = 4.39, *p* < .001) and night-shift nurses vs. evening-shift nurses (*B* = 3.95, *p* < .001) were positive, which means that night-shift nurses were under significantly greater stress than day-shift and evening-shift nurses. With regard to CARi, the regression coefficients of night-shift nurses vs. day-shift nurses (*B* = −3.41, *p* < .001) and night-shift nurses vs. evening-shift nurses (*B* = −2.92, *p* < .01) were negative, which means that night-shift nurses ha**ve** significantly lower CARi values than day-shift and evening-shift nurses. With regard to cortisol levels 30 minutes after waking, the regression coefficients of night-shift nurses vs. day-shift nurses (*B* = −3.88, *p* < .01) and night-shift nurses vs. evening-shift nurses (*B* = −3.31, *p* < .01) were negative, which means that night-shift nurses ha**ve** significantly lower cortisol levels 30 minutes after waking than day-shift and evening-shift nurses. These results indicate that female night-shift nurses display the lowest CARi and cortisol levels 30 minutes after waking and are more negatively affected by being unable to complete personal tasks.

## 1. Introduction

The work environments and duties of nursing personnel are complex. They must monitor the mental and physical conditions of their patients, meet the needs of the family members of their patients, and communicate with their supervisors, coworkers, and other medical personnel. In addition to their work environments being full of noise and communicable diseases, they must also cope with heavy workloads and time stress. These are all sources of stress in nursing [1].

Researchers have identified that shift work itself is a stressor. Nurses working shifts must continuously adjust to changes in the external environment, which means ever-changing lifestyles and psychological stress [2]. Altering the time of waking can have a profound impact on cortisol levels, which are a crucial activity index of neuroendocrine systems faced with stress [3]. Cortisol secretion is a physiological self-defense mechanism to cope with stress [4]. HPA axis stimulation has a stronger impact on hormones when it takes place in the morning than when it happens in the evening [5]. Thus, sleep schedules are closely associated with cortisol levels. Balbo et al. also indicted that working at night results in lower cortisol levels in the morning after, but after the fifth night of working at night, cortisol levels will become the inverse of the original day-night cycle [6].

The cortisol awakening response (CAR) plays a crucial role in human HPA axis activation during the awakening process. It is an important indicator of HPA axis activity and is highly associated with the arousal system in the brainstem [7]. CAR is widely accepted as being fairly stable; cortisol levels in the human body swiftly rise upon wakening and peaks 30 minutes later. At present, it is known to be associated with energy increase or anticipated stress, which elevates average cortisol levels by 50% to 100%. Such levels persist for at least an hour and then slowly decline. Subsequently, secretions decline from day to night and maintain periodic changes [8, 9].

**Figure 1:**
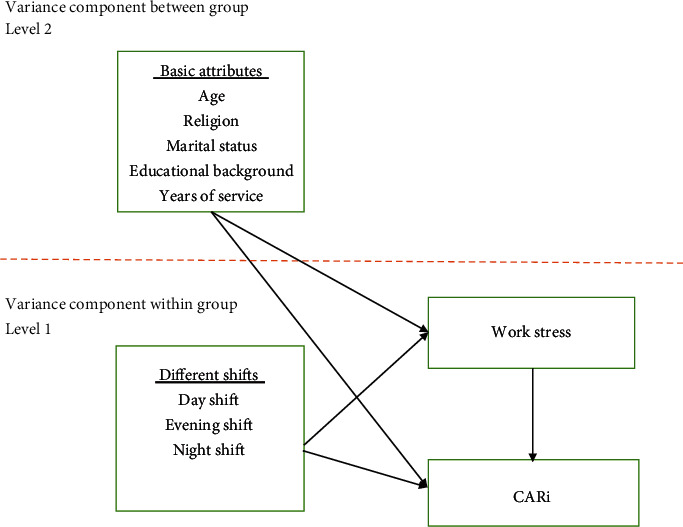
Research framework.

Although an increasing number of studies are using CAR to detect adrenocortical activity, most studies on nursing personnel working shifts examined stress and CAR on a single level. No relevant studies have used multilevel models so far. The hierarchical linear model (HLM) is currently one of the most suitable statistical methods for multilevel data analysis. The model regards observation timepoints as the first level and the basic attributes of nursing personnel as the second level while examining the relationships among variables. We thus performed a longitudinal analysis on the panel data collected and used the HLM growth model to (1) understand the changes in CAR and work stress in nursing personnel working in different shifts, (2) determine the influence of specific attributes of female nurses on CAR and work stress, and (3) examine the relationship between CAR and stress.  Figure 1 displays the conceptual framework of this study.

## 2. Materials and Methods

### 2.1. Study Design and Sample

The presented study was a component of a large-scale project investigated nurses working shifts at a teaching hospital in Northern Taiwan. Preliminary findings of this project have been previously reported [10]. We recruited 41 nurses who were working monthly rotating shifts between the ages of 20 and 45 with more than one year of experience as a clinical nurse. Nurses who were pregnant or were using contraceptives or hormone medication were excluded. Monthly rotating shifts were defined as the three-shift system in which working hours are divided into day shift (08:00-16:00), evening shift (16:00-24:00), and night shift (00:00-08:00). Nurses change shifts every month, which is classified as slow rotation [11].

### 2.2. Procedure

We adopted a prospective, longitudinal research design with purposive sampling. Repeated measurements were performed on shift nurses to determine the influence of different shifts and work stress on CAR. With the approval of the institutional review board (IRB), we explained our objectives and procedure to the research participants and gained their consent. Wirth et al. found that individuals who sometimes work night shift or evening shift wake up with lower CAR values than those who permanently work day shift [12]. The difference between these two groups of individuals is greatest on the 5th day after a shift change and gradually decreases from the 7th day to the 14th day after a shift change. To eliminate the influence of shift changes, we conducted the first structured interviews 14 days after shift changes. Subsequent structured interviews were then conducted on the 14th day of the next month and the 14th day of the following month for follow-up data collection. Our procedure follows nurses across three consecutive shift changes over three months that cover day, evening, and night shifts.

### 2.3. Measures

The measurement tools included a basic information sheet, the Taiwan Nurse Stress Checklist (NSC) and a radioimmunoassay (RIA):

Basic information sheet: This included information on the age, religion, marital status, educational background, and years of service of the nurses.

Taiwan Nurse Stress Checklist (NSC): The Taiwan NSC is based on the scale developed by Benoliel to gauge the work stress that a nurse has experienced during the week prior [13]. Each question item was measured using a nine-point Likert scale ranging from 0 to 8 points, with a higher score indicating greater stress. Tsai and Chen revised the scale for application in Taiwan [14]. The checklist includes four aspects of stress containing a total of 43 question items. Personal reactions contain 16 question items with regard to the negative physical and mental reactions of the nurses toward their work. Work concerns contain 13 question items regarding issues in their communication with patients, patient family members, or doctors during work as well as their personal disappointed expectations of the professional or healthcare system. Work competence contains 11 question items concerning self-satisfaction with work completion and personal professional abilities. Inability to complete personal tasks contain 3 question items regarding the interactions between the nurses and their living environment. The Cronbach's *α* of each aspect was greater than 0.84.

Radioimmunoassay (RIA): In this study, we used RIA to measure the amount of cortisol in the saliva of the participants. A single saliva sample presents inadequate stability in cortisol results, so saliva samples were collected for three consecutive days at two different times to measure CAR variations, one immediately upon waking and the other at 30 minutes after waking [15]. CARi (the increase in cortisol following waking) is an extremely valid and standardized method of measuring HPA axis responsiveness and estimates the average increase in cortisol levels during the first 30 minutes after waking [9]. For the sake of stability in the cortisol saliva sample data, the participants were asked not to eat within the 30 minutes before taking their sample and collect more than 3 ml of saliva. All saliva samples had to be frozen for storage after being taken. Prior to analysis, the samples were stored in a freezer at -20°C. System precision tests on the cortisol analysis system were performed five times as suggested by Wilson and Miles [16]. The intra-assay CV % and interassay CV % of the cortisol RIA analysis system were 0.96% and 12.09%, respectively, and the detection limit was 1.66 nmol/l.

### 2.4. Data Analysis

We first employed SPSS for Windows 25.0 (SPSS, Chicago, IL, USA) to obtain the descriptive statistics and subsequently performed an ANOVA. Using HLM 6.03 (SSI, 2010), we then conducted HLM analysis on the panel data obtained. In model selection, we first used the null model to derive within group variance (*σ*2) and between group variance (*τ*00). According to Cohen, an intraclass correlation coefficient (ICC), which equals (*τ*00)/(*τ*00 + *σ*2), greater than 5.9% (moderate effects) indicates significant variance in the result variables of individual participants [17]. We used a random coefficients regression model and an intercepts-as-outcomes model for model validation. If the regression coefficient *B* has significant meaning (*p* > .05), the basic attribute or shift of the nurses (independent variable) exert a significant impact on their work stress or CARi (dependent variable).

## 3. Results

### 3.1. Basic Attributes of Female Shift Nurses

The mean age of the participating nurses was 25.0 (range = 21.0 − 40.0). In terms of religion, the largest group (48.8%) was not religious. The vast majority of the participants (95.1%) were either single or divorced. Most (70.7%) had a bachelor's degree or higher. With regard to years of service, the largest group (43.9%) has worked for 1-3 years (Table 1).

### 3.2. CAR Differences among Shifts

**Figure 2:**
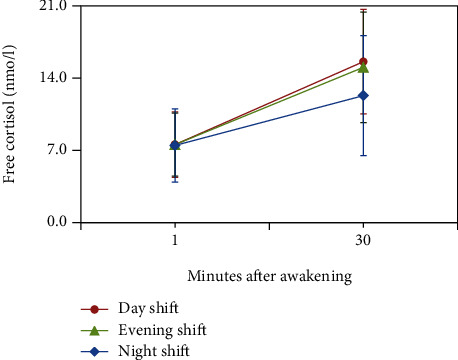
Mean cortisol awakening responses and standard deviation of 41 female nurses working different shifts.

The CARi values of the nurses in morning, evening, and night shifts were 8.04 (SD = 4.26), 7.49 (SD = 4.28), and 4.83 (SD = 3.60). An ANOVA revealed CARi differences among different shifts (*F* (2, 120) = 7.13, *p* = .001).  Figure 2 presents the CARi trends in the nurses working different shifts.

Level-1 (intraindividual) variance of the CARi, cortisol levels upon waking, and cortisol levels 30 minutes after waking was 12.42, 4.91, and 15.75, respectively, whereas Level-2 (interindividual) variance was 5.87 (*p* < .05), 5.71 (*p* < .001), and 15.45 (*p* < .001), respectively. For the different shifts, the ICCs were 0.3208, 0.5379, and 0.4952, respectively, all higher than the threshold of 13.8% for high correlation [17]. This means that the interclass differences among the nurses of different shifts in CARi, cortisol levels upon waking, and cortisol levels 30 minutes after waking cannot be ignored and that cross-level analysis is necessary [18]. Thus, a respective 32.08%, 53.79%, and 49.52% of the total variance in CARi, cortisol levels upon waking, and cortisol levels 30 minutes after waking are caused by interindividual variance.

As shown in Table 2, significant differences existed between the CARi of night-shift nurses and that of day-shift nurses (*B* = −3.41, *p* < .001), and between the CARi of night-shift nurses and evening-shift nurses (*B* = −2.92, *p* < .01), indicating that night-shift nurses exhibited significantly lower CARi than did day-shift nurses or evening-shift nurses. The other variables did not have a significant impact on CARi and no variables were shown to have a significant impact on cortisol levels upon waking, whereas significant differences existed for cortisol levels 30 minutes after waking between night-shift nurses and day-shift nurses (*B* = −3.88, *p* < .001), and between night-shift nurses and evening-shift nurses (*B* = −3.31, *p* < .01), indicating that night-shift nurses exhibited significantly lower cortisol levels 30 minutes after waking than did day-shift nurses or evening-shift nurses. The other variables did not have a significant impact on cortisol levels 30 minutes after waking.

### 3.3. Work Stress Differences among Shifts

The work stress scores of the nurses in morning, evening, and night shifts were 113.41 (SD = 64.35), 119.17 (SD = 58.94), and 125.46 (SD = 57.72), respectively. An ANOVA revealed no significant differences in work stress among the nurses working different shifts (*F* (2, 120) = 0.41, *p* = 0.666).

Level-1 (intraindividual) variance of the work stress of the female shift nurses was 843.93, while Level-2 (interindividual) variance was 2815.89 (*p* < .001). The ICC was 0.7694, indicating that differences in work stress among the nurses of different shifts cannot be ignored and that 76.94% of the total variance is caused by interindividual variance.

As can be seen in Table 3, the regression coefficients of all of the variables did not reach the level of significance in personal reaction and overall work stress. For work concerns, only age (*B* = −3.25, *p* < .05) reached the level of significance, indicating that older nurses derived less stress from work concerns. With respect to the inability to complete personal tasks, significant differences existed between night-shift nurses and day-shift nurses (*B* = 4.39, *p* < .001), and between night-shift nurses and evening-shift nurses (*B* = 3.95, *p* < .001), thus night-shift nurses derived significantly more stress from the inability to complete personal tasks than did day-shift nurses or evening-shift nurses.

### 3.4. Influence of Work Stress on CAR

An ANOVA of the stress from personal reactions, work concerns, work competence, and inability to complete personal tasks for nurses working the three different shifts presented no significant differences. With the shift and other personal attributes controlled, the four aspects of work stress also showed no significant differences in their influence on the CARi, cortisol levels upon waking, or cortisol levels 30 minutes after waking of female shift nurses (Table 3).

## 4. Discussion

Waking time and stress are both considered factors influencing CAR, but the correlations with these factors are still unclear. This study is the first to adopt a longitudinal approach to examine the differences among women working different shifts (and waking up at different times) and their correlations with CAR and work stress. The study results revealed that after the personal attributes of the nurses were controlled, night-shift nurses displayed significantly lower CARi and cortisol levels 30 minutes after waking than did day-shift nurses or evening-shift nurses. When basic attributes were controlled, the results showed that older nurses derived less stress from work concerns. Following the control of the personal attributes of the nurses, the results indicated that the night-shift nurses derived more stress from the inability to complete personal tasks than did day-shift nurses or evening-shift nurses.

Cortisol plays a crucial role in regulating the coping mechanism of chronic stress. Shift work exerts influence on multiple physiological, neuroendocrine, and hormonal functions. As a result, CAR is currently considered to be a major index of shift work tolerance [19]. Research has shown that changes in CAR may be the result of increased stress and impaired sleep quality following shift changes; however, controlling for shift factor revealed no significant differences in CAR variations [20]. Many existing studies presented different findings. Federenko et al. found significant increase in CAR in day-shift nurses than in evening-shift or night-shift nurses [21]. Niu et al. further explained that working night shifts conflicts with the sleep schedule of individuals, which causes circadian rhythm disorders, affects sleep quality, induces fatigue, and breaks concentration, thereby reducing work efficiency [22]. Night-shift workers sleep during the day when their cortisol levels are high, which affects the quality and duration of sleep. On average, they sleep 1 to 4 hours less than do day-shift workers sleep during the night, leading to high sleep debts that can cause chronic fatigue. In this study, we performed measurements on the 14th day after shift changes as suggested by Wirth et al. to control the influence of shift change. Interestingly, significant CAR differences still existed [12]. This result supports that CAR is associated with waking time. Individuals who wake in the morning have significantly higher cortisol levels. Those who work evening or night shift (sleep during the day) displayed significantly weaker CAR than those who permanently work day shift (sleep during the night), likely due to the normal circadian rhythm of the latter group [23, 24].

The results of this study also revealed that work stress which shift nurses must cope with varies with age, with older shift nurses feeling less stress from work concerns. Nursing personnel work on the front lines and must have good professional decision-making and execution abilities, be skilled, and have good relationships with patients and other medical personnel, all of which come with increased work experience, because they come into direct contact with patients and their family members [25].

Furthermore, the results of this study also indicated that night-shift nurses suffer more stress from their inability to complete personal tasks. This finding is consistent with the results of other studies. The sleeping period of night-shift nurses is often during the early night and likely when their friends or family have free time, thereby preventing them from spending time with their friends and family. Their shift work causes stress on the best way to utilize their time and hinders them from completing personal tasks. It is difficult for them to find the time for social activities and family life, which are the primary causes of greater stress [26].

Stress the affect CAR magnitudes. Many studies have indicated that CAR is an indicator of stress or emotional disorders. Increased CAR helps the body cope with the stresses in life [3, 27]. A meta-analysis conducted by Chida and Steptoe revealed that CAR is positively correlated with work stress and life stress and negatively correlated with fatigue or burnout [28]. Garcia et al. further pointed out that only individuals under a moderate or severe amount of stress perceive themselves as having poorer health and that those with poorer self-perceived health work up with significantly reduced CAR and smaller increases in cortisol levels after waking up [29]. Thus, only moderate and severe amounts of stress and poor self-perceived health alter CAR, which means that a low level of stress does not affect CAR or self-perceived health. This is consistent with the results of our study. After the personal attributes and shifts of the nurses were controlled, we found that the various aspects of work stress did not significantly affect CARi. One explanation for this observation is that the month-long tracking periods of the participants may not be long enough for any significant changes to appear in the various aspects of work stress, so indeed, CARi would not be affected.

The CAR is regarded as the body's preparation for the work and challenges of the day to come. It helps maintain homeostasis and promote adaptive responses [30]. This study found that nurses working monthly rotating shifts have lower CARi and cortisol levels 30 minutes after waking when they work the night shift than when they work the day shift or the evening shift and that they derive more stress from the inability to complete personal tasks. These results indicate that when working the night shift, nurses have difficulty adapting their biological clocks and personal lives. We therefore suggest that night shifts do not continue for too long so as to give the nurses adequate rest and enable them to complete personal tasks, which will in turn prevent long-term sleep deprivation from harming their health and affecting their work performance and emotions [31].

The number of samples collected in this study was limited and only female nurses at a single hospital were investigated, so the results may not be applicable to other hospitals, other departments, or male nurses. CAR changes were based on only two timepoints: upon waking and 30 minutes after waking. It is suggested that future studies include three or more timepoints to measure the changes in CAR which could lead to a more comprehensive analysis. A number of other factors also influence cortisol production, including sleep quality, exercise, weight loss diets, coffee intake, and ovulation time. However, with subjective research conditions, this study could only exclude nurses who were pregnant or using contraceptives, which was the greatest limitation of this study.

## 5. Conclusion

The results of this study indicated that female night-shift nurses displayed the lowest CARi and cortisol levels 30 minutes after waking and were more troubled with the inability to complete personal tasks. Our results imply that monitoring cortisol levels may be necessary to understanding the HPA axis variations in nurses that work shifts. These results also provide hospital managers with a reference when arranging nurse shift schedules. Suitable shift arrangements could reduce the impact of shift work on female nurses and their health.

## Figures and Tables

**Table 1:** Basic information of shift nurses.

Characteristics	*n*	Value (%)

Religion		
None	20	48.8
Buddhism/Taoism	17	41.5
Christianity/Catholicism	4	9.8
Marital status		
Married	2	4.9
Single or divorced	39	95.1
Educational background		
Junior college	12	29.3
University or above	29	70.7
Years of service		
1-3 years	18	43.9
3-5 years	9	22.0
5-7 years	8	19.5
Over 7 years	6	14.6

	Median	Range
Age (years)	25	21-40

**Table 2:** Influence of research variables on work stress of shift nurses.

Independent variables	Personal reaction	Work concerns	Work competence	Inability to complete personal tasks	NSCG total score

Fixed effect					
Level 1					
Intercept	63.03^∗^	87.49^∗∗^	63.37^∗^	7.18	221.79^∗∗^
Shift					
Evening vs day	2.91	3.55	-1.30	0.44	5.61
Night vs day	5.25	1.95	0.31	4.39^∗∗∗^	11.91
Night vs evening	2.34	-1.61	1.61	3.95^∗∗∗^	6.29
Level 2					
Age	-1.55	-3.25^∗^	-1.29	-0.04	-6.17
Religion					
Buddhism/Taoism^a^	8.86	6.93	4.53	1.57	21.88
Christianity/Catholicism^a^	-4.44	-10.49	-6.59	0.90	-20.70
Marital status (single or divorced) ^b^	7.63	12.66	0.98	0.52	21.98
Educational background (university or above) ^c^	9.95	6.10	1.31	1.70	19.16
Years of service					
3-5 years ^d^	8.52	8.13	2.55	0.15	19.47
5-7 years ^d^	6.97	17.17	-0.84	0.23	23.72
Over 7 years ^d^	-7.94	28.39	4.34	-2.96	22.35

Random effect					
Level 2 (variance component between groups)	571.72^∗∗∗^	345.46^∗∗∗^	227.96^∗∗∗^	17.97^∗∗^	2930.95^∗∗∗^
Level 1 (variance component within group)	180.57^∗∗∗^	88.09^∗∗∗^	121.17^∗∗∗^	17.16^∗∗∗^	821.90^∗∗∗^

^∗^ *p* < .05; ^∗∗^*p* < .01; ^∗∗∗^*p* < .001. ^a^ Religion as the reference group. ^b^ Married as the reference group. ^c^ Junior college as the reference group. ^d^ 1-3 years of service as the reference group.

**Table 3:** Influence of study variables on CAR.

Independent variables	CARi	Cortisol levels upon waking	Cortisol levels 30mins after waking

Fixed effect			
Level 1			
Intercept	11.02	3.96	15.16^∗^
Shift			
Evening vs day	-0.50	-0.06	-0.56
Night vs day	-3.41^∗∗^	-0.52	-3.88^∗∗^
Night vs evening	-2.92^∗∗^	-0.45	-3.31^∗∗^
Work stress			
Personal reactions	-0.02	0.01	-0.01
Work concerns	-0.01	-0.01	-0.02
Work competence	0.01	-0.03	-0.02
Inability to complete personal tasks	0.07	0.09	0.15
Level 2			
Age	-0.18	-0.01	-0.19
Religion			
Buddhism/Taoism^a^	0.38	0.53	0.93
Christianity/Catholicism^a^	0.24	-0.67	-0.42
Marital status (single or divorced) ^b^	0.99	3.22	4.22
Educational background (university or above) ^c^	1.19	0.53	1.74
Years of service			
3-5 years ^d^	-0.57	0.77	0.20
5-7 years ^d^	0.14	0.44	0.56
Over 7 years ^d^	0.33	-0.09	0.19

Random effect			
Level 2 (variance component between groups)	8.84^∗∗^	6.03^∗∗^	18.99^∗∗^
Level 1 (variance component within group)	9.99^∗∗^	5.03^∗∗∗^	13.11^∗∗∗^

^∗^ *p* < .05; ^∗∗^*p* < .01; ^∗∗∗^*p* < .001. ^a^ Religion as the reference group. ^b^ Married as the reference group. ^c^ Junior college as the reference group. ^d^ 1-3 years of service as the reference group.

## Data Availability

The statistical data used to support the findings of this study are available from the corresponding author upon request.
